# Cost-effectiveness of psychosocial assessment for individuals who present to hospital following self-harm in England: A model-based retrospective analysis

**DOI:** 10.1192/j.eurpsy.2022.5

**Published:** 2022-01-31

**Authors:** David McDaid, A-La Park, Apostolos Tsiachristas, Fiona Brand, Deborah Casey, Caroline Clements, Galit Geulayov, Nav Kapur, Jennifer Ness, Keith Waters, Keith Hawton

**Affiliations:** 1 Care Policy and Evaluation Centre, Department of Health Policy, London School of Economics and Political Science, London, United Kingdom; 2 Health Economics Research Centre, Nuffield Department of Population Health, University of Oxford, Oxford, United Kingdom; 3 Centre for Suicide Research, Department of Psychiatry, Warneford Hospital, University of Oxford, Oxford, United Kingdom; 4 Oxford Health NHS Foundation Trust, Oxford, United Kingdom; 5 Centre for Mental Health and Safety, University of Manchester, Manchester, United Kingdom; 6 Greater Manchester Mental Health NHS Foundation Trust, Manchester, United Kingdom; 7 NIHR Greater Manchester Patient Safety Translational Research Centre, University of Manchester, Manchester, United Kingdom; 8 Centre for Self-Harm and Suicide Prevention Research, Derbyshire Healthcare NHS Foundation Trust, Derby, United Kingdom

**Keywords:** Economic issues, emergency departments, health economics, health services research, suicide

## Abstract

**Background:**

Guidance in England recommends psychosocial assessment when presenting to hospital following self-harm but adherence is variable. There is some evidence suggesting that psychosocial assessment is associated with lower risk of subsequent presentation to hospital for self-harm, but the potential cost-effectiveness of psychosocial assessment for hospital-presenting self-harm is unknown.

**Methods:**

A three-state four-cycle Markov model was used to assess cost-effectiveness of psychosocial assessment after self-harm compared with no assessment over 2 years. Data on risk of subsequent self-harm and hospital costs of treating self-harm were drawn from the Multicentre Study of Self-Harm in England, while estimates of effectiveness of psychosocial assessment on risk of self-harm, quality of life, and other costs were drawn from literature. Incremental cost-effectiveness ratios (ICERs) for cost per Quality Adjusted Life Year (QALY) gained were estimated. Parameter uncertainty was addressed in univariate and probabilistic sensitivity analyses.

**Results:**

Cost per QALY gained from psychosocial assessment was £10,962 (95% uncertainty interval [UI] £15,538–£9,219) from the National Health Service (NHS) perspective and £9,980 (95% UI £14,538–£6,938) from the societal perspective. Results were generally robust to changes in model assumptions. The probability of the ICER being below £20,000 per QALY gained was 78%, rising to 91% with a £30,000 threshold.

**Conclusions:**

Psychosocial assessment as implemented in the English NHS is likely to be cost-effective. This evidence could support adherence to NICE guidelines. However, further evidence is needed about the precise impacts of psychosocial assessment on self-harm repetition and costs to individuals and their families beyond immediate hospital stay.

## Introduction

Self-harm, defined as nonfatal intentional self-poisoning or self-injury, irrespective of degree of suicidal intent or other motives [1,2], is a major health care problem globally. In England, it involves over 200,000 hospital presentations at a cost of £128 million annually [3,4]. In addition to these immediate hospital costs, there will be other substantial costs both to health systems and wider society [5]. Risk of completed suicide is more than 50 times greater in people who present to hospital for self-harm than the general population and especially high in the months following hospital discharge [6]. Past studies highlight very high lifetime costs of premature mortality [7,8].

Policymakers have also examined the economic case as part of the development of English national guidelines on suicide prevention [9,10]. There is therefore an economic, as well as moral imperative, to understand not only what works in preventing self-harm and suicide, but also the cost-effectiveness of interventions.

One potential intervention is psychosocial assessment, which is recommended for all hospital presenting self-harm in England [2,11]. On average, around 50–60% of people presenting to accident and emergency (A&E) departments for self-harm receive a psychosocial assessment, although this proportion varies across sites [12,13]. Typically carried out by psychiatric liaison staff, this includes assessing patients’ problems, mental state, risk factors and needs, and arranging appropriate aftercare. Investment in greater adherence to guidance on use of psychosocial assessment may help reduce risk of repeat self-harm [14], as well as having other potential therapeutic benefits.

It may therefore be a cost-effective mechanism from a public health perspective but there is relatively little evidence on the economic case for increasing adherence to guidance.

This study aimed to model the potential cost-effectiveness of psychosocial assessment for hospital presenting self-harm in England compared to no assessment. The work draws on data on hospital presenting self-harm from the Multicentre Study of Self-Harm in England (MSH). This systematically collects data, captured via clinicians and clinical records for all hospital presentations for self-harm in Oxford, Derby, and Manchester. These areas have diverse populations with varied sociodemographic characteristics, and may therefore, provide a reasonably representative picture of self-harm in England [3].

## Methods

Health economic modelling studies are widely used to help determine the potential strength of the economic case for action [15]. Models bring together evidence on effectiveness, resource use, and costs from multiple sources. One of the principal approaches is Markov modelling. It can be used to model uncertain processes over multiple time periods known as cycles and reflect circumstances where individual health and outcomes can fluctuate [16]. Markov modelling has been used by public health agencies in England to support local decision makers, for instance, to develop their mental health promotion and disorder prevention strategies, including work to prevent bullying in schools and suicide in adults [17].

**Figure 1.**
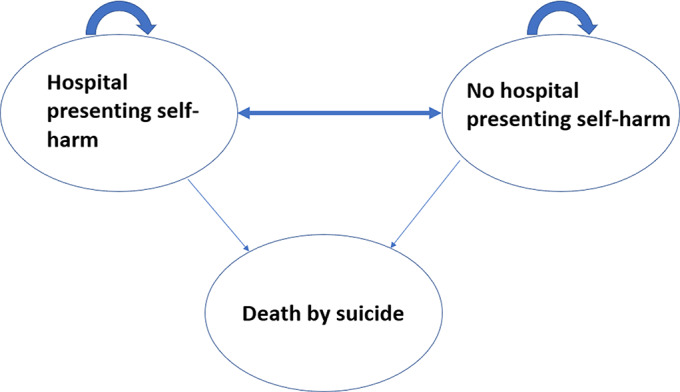
Overview of three-state Markov model.

A three-state Markov model has been constructed. The model runs over 2 years with four Markov cycle time periods in total, each lasting 6 months, comparing receipt of psychosocial assessment following each hospital presenting self-harm event to nonreceipt of psychosocial assessment after a self-harm event. Figure 1 provides an overview of the model’s health states, with a more detailed excerpt shown in Supplementary Figure S1. Individuals enter the model when initially presenting at a hospital A&E department following a self-harm event. They may be treated in A&E only or admitted to hospital for treatment and observation. Patterns of treatment and length of stay also vary depending on physical severity of self-harm. The model assumes that in each subsequent cycle there are three possible states: no hospital-presenting self-harm event, a further A&E presentation following self-harm, and death from suicide.

The primary outcome is quality of life. Utility values are assigned to the three health states and Quality Adjusted Life Years (QALYs) are estimated based on time spent in each health state. Mean costs associated with self-harm events in each cycle were computed. All costs and outcomes are reported in 2020 British pounds (£), discounted at a recommended annual rate of 3.5% after 1 year (i.e., the last two 6-month cycles) [18]. Where necessary, costs have been converted to 2020 prices using GDP Deflators [19]. Our primary analysis is conducted from the publicly funded English National Health Service (NHS) perspective, but we also report results from a societal perspective taking account of productivity losses to patients when in hospital. No other productivity losses, such as impacts on families or premature death through suicide, are included. Cumulative QALYs and total costs over the model’s 2-year time horizon have been calculated and incremental costs per QALY gained estimated. A CHEERS (Consolidated Health Economic Evaluation Reporting Standards) recommended reporting checklist for health economic studies [20] is included in Supplementary Table S1.

### Sensitivity analysis and uncertainty

Sensitivity analyses were conducted to assess robustness of results to underlying input parameters and assumptions. In univariate sensitivity analysis, we varied most individual parameters in the NHS perspective model, one at a time, by up to 20% from their mean values. One exception was probability of repeat self-harm after psychosocial assessment. In this case, the variation was greater to reflect conservative values reported in literature [21–23].

Additionally, we looked at the possible direct impact of psychosocial assessment on suicide. In our baseline model, we assumed no risk reduction, given a lack of evidence [24]; here we examined the impact of risk reduction between 0 and 100%. We further looked at the impact of increasing the value of productivity losses up to £100 per hospital inpatient day.

We also conducted Monte-Carlo simulation, where uncertainty associated with model variables can be estimated from the parameters’ distribution. This is done by randomly sampling values for selected parameters from within their distributions simultaneously and then calculating incremental cost-effectiveness. We repeated this exercise 10,000 times. Following best practice, input parameters were assigned beta, gamma, or log-normal shaped distributions, as appropriate [25]. We visually show results on cost-effectiveness planes, where all 10,000 combinations of incremental costs and incremental QALYs were plotted. Additionally, histograms showing distribution of net monetary benefits (NMB) were constructed. The value of each QALY in NMB was assumed to be £20,000, equivalent to the notional willingness to pay threshold per QALY gained used by NICE when making recommendations on the reimbursement of health system interventions [26].

Cost-effectiveness acceptability curves, indicating likelihood of the intervention being considered cost-effective at different thresholds of willingness to pay per QALY gained were generated from NHS and societal perspectives. All analyses are modeled using TreeAge Pro software (TreeAge Software, LLC, Williamstown, MA).

### Model parameters

Model parameters and assumptions on distributions in probabilistic sensitivity analysis (PSA) are shown in Table 1. The probability of repeat self-harm in the next model cycle falls from 0.18 [27] to 0.11 after psychosocial assessment, based on observed experience in two of the three hospital groups covered by the MSH where the relative risk of self-harm following psychosocial assessment was 0.59 of the risk without assessment [14].

**Table 1.** Model parameters.

Input parameter	Deterministic value	Distribution in PSA	Source
**Self-harm and suicide risks**			
Risk of repeat self-harm in next cycle with psychosocial assessment	0.106	Beta	[14]
Risk of repeat self-harm in next cycle without psychosocial assessment	0.180	Beta	[27]
Risk of suicide in next cycle following self-harm	0.007	Beta	[6]
Risk of suicide in next cycle if no self-harm	0.003	Beta	[6]
**Utility values**			
Self-harm event in cycle	0.68	Beta	[28]
No self-harm event in cycle	0.929	Beta	[29]
Death through suicide in cycle	0	Fixed	[30]
**Unit costs**			
Ambulance (per visit): National tariff see, treat and convey tariff	£261	Gamma	[31]
Psychosocial assessment (adult)	£243	Gamma	[27]
Psychosocial assessment (adolescent)	£417	Gamma	[27]
Poisoning (no admission)	£255	Gamma	[27]
Poisoning (admission)	£703	Gamma	[27]
Self-injury (no admission)	£123	Gamma	[27]
Self-injury (admission)	£1,188	Gamma	[27]
Combined poisoning and self-injury (no admission)	£394	Gamma	[27]
Combined poisoning and self-injury (admission)	£901	Gamma	[27]
Productivity loss (living wage per day)	£65.40	Gamma	[32]
Length of stay by type of self-harm (days)			
Poisoning	1.03	Gamma	[27]
Self-injury	1.54	Gamma	[27]
Combined poisoning and self-injury	1.00	Gamma	[27]
**Other probabilities**			
Arrival at A&E by ambulance	0.21	Beta	[33]
A&E attendance: poisoning	0.70	Beta	[27]
A&E attendance: self-injury	0.22	Beta	[27]
A&E attendance: combined poisoning and self-injury	0.08	Beta	[27]
Hospital admission: poisoning	0.88	Beta	[27]
Hospital admission: self-injury	0.49	Beta	[27]
Hospital admission: combined poisoning and self-injury	0.84	Beta	[27]
Discount rate (after 12 months)	0.035	Fixed	[18]

The risk of suicide in the next model cycle also draws on analysis of 13 years of self-harm hospital presentations in the MSH. Risk remained 55 times greater than that of the general population after 12 months [6]. Given the lack of evidence, we assumed psychosocial assessment had no direct impact on suicide, but changed this assumption in univariate sensitivity analysis.

On average 21% of A&E attendances arrive by ambulance [33]. We assumed this applied to self-harm patients, and applied a national tariff to costs [31]. Average costs per psychosocial assessment were taken from detailed analyses in hospitals in Oxford and Derby that are part of the MSH [4,27]. This average cost reflects higher costs of assessments for under-18s. Mean treatment costs for self-harm are also taken from previous analyses of costs for admitted and non-admitted patients in MSH Oxford and Derby hospitals. Patterns of self-harm: poisoning or self-injury only or a combination of the two methods, and likelihood of hospital admission following self-harm are also based on rates in the MSH. In the societal perspective analysis, we also assumed a full day of productivity loss for each day of an inpatient stay, based on the average length of stay associated with different injuries. We assumed each productivity loss day would be 7.5 hours, valued at the national living wage rate [32].

Limited information is available on the quality of life impacts of self-harm, for both adults and adolescents. Previous economic evaluations of self-harm prevention have tended to use quality of life weights associated with specific mental disorders such as depression. Recently, quality of life data on self-harm in 754 adolescents in England were collected using the EQ-5D-3L, an instrument widely used to elicit health-related quality of life values, as part of a trial of family therapy [28]. In line with this trial, we have assumed that each cycle where an individual self-harms will have a quality of life weight (or utility) of 0.68. So, in a single cycle, there are on average 0.34 QALYs associated with experiencing self-harm, with 1.36 QALYs if self-harming in each cycle of the model’s 2-year time horizon. Following usual practice, death was set to a value of 0 [30] while the quality of life weight in cycles without self-harm was assumed equivalent to population norms for adolescents and young adults in England of 0.929 [29]. In sensitivity analysis, we examine the impact of lowering this quality of life weight down to 0.80 given the potential for enduring chronic poor mental health in some people who self-harm [34].

## Results

From an NHS perspective adherence to guidance on psychosocial assessment would potentially be cost-effective with a cost per QALY gained of £10,962 (95% uncertainty interval [UI], £15,538–£9,219) (Table 2). When immediate productivity losses restricted only to inpatient time in hospital are also included, incremental cost per QALY gained falls to £9,980 (95% UI, £14,538, £6,938). These are below the notional £20,000 cost per QALY gained threshold that is one of the factors taken into account in deliberations by NICE before making recommendations on the reimbursement of health technologies and public health interventions.

**Table 2.** Incremental cost-effectiveness results.

	Psychosocial assessment	No psychosocial assessment	Incremental cost and effect
NHS perspective			
Cost (95% CI)	£1,223 (£1,086, £1,372)	£977 (£884, £1,077)	£246 (£202, £295)
QALYs (95% CI)	1.253 (1.085, 1.418)	1.231 (1.072, 1.386)	0.022 (0.013, 0.032)
ICER (95% UI)	£10,962 (£15,538, £9,219)		
Societal perspective			
Cost (95% CI)	£1,377 (£1,197, £1,637)	£1,153 (£1,009, £1,411)	£224 (£189, £222)
QALYs (95% CI)	1.253 (1.085, 1.418)	1.231 (1.072, 1.386)	0.022 (0.013, 0.032)
ICER (95% UI)	£9,980 (£14,538, £6,938)		

Abbreviations: CI, confidence interval; ICER, incremental cost-effectiveness ratio; NHS, National Health Service; QALY, Quality Adjusted Life Year; UI, uncertainty interval.

### Univariate sensitivity analysis

**Figure 2.**
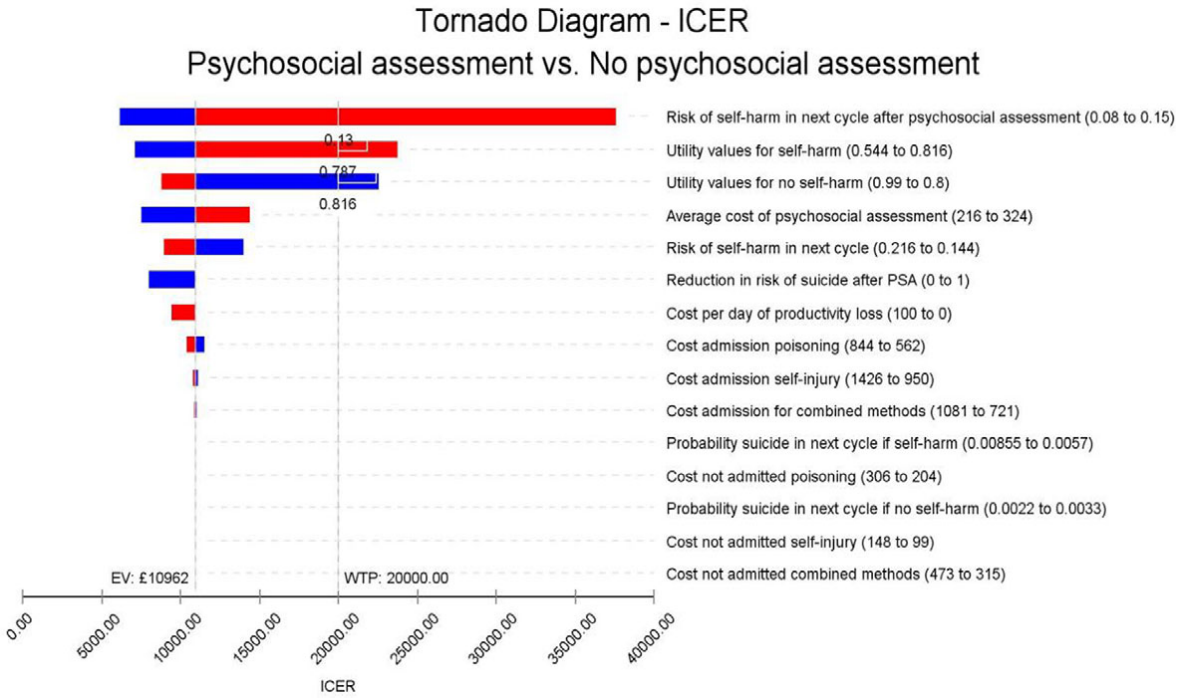
Tornado diagram. The vertical line shows the mean expected ICER of £10,887 per QALY gained in our base case NHS perspective scenario. Red bar segments indicate that the value of each parameter has increased, while blue segments show parameter values have fallen. Values to the right of the vertical base case scenario line indicate less favourable cost-effectiveness with the cost per QALY increasing compared to the base case scenario, while those to the left indicate an improvement in cost-effectiveness, with the cost per QALY gained reducing. The more sensitive a model parameter is, the higher it is in the diagram, thus risk of self-harm after psychosocial assessment is the most sensitive parameter in the diagram.

The sensitivity of the incremental cost-effectiveness ratios (ICERs) to individual model parameters are shown as a “Tornado” diagram in Figure 2. This figure shows sensitivity of results to each parameter in a hierarchical order (i.e., the parameter with greatest sensitivity at the top). The figure indicates results are most sensitive to changes in assumptions on likelihood of repeat self-harm following psychosocial assessment. If this risk increases from the baseline value of 0.106–0.130 then the cost per QALY value breaches the £20,000 QALY threshold. If risk of self-harm increased further to 0.15, in line with the most conservative estimate of effect reported in an English study [21], then the cost per QALY would increase to £37,633. The analysis also revealed if utility values for self-harm were above 0.787 or if values for no self-harm fall below 0.816 then cost per QALY is above the £20,000 threshold. Conversely, if utility values for self-harm were 20% lower than in our baseline model, then the cost per QALY gained became more favourable at £7,122.

If the average cost of psychosocial assessment were to rise by 20% then the incremental cost per QALY would rise to £14,291, or conversely, a similar fall would mean a cost per QALY of £7,484. Assumptions on likelihood of repeat self-harm also have some impact, leading to values of £13,894 and £8,883 following a 20% decrease or increase in repeat self-harm risk in any cycle.

If any direct impact between psychosocial assessment and reduced risk of suicide could be established, this is likely to only have a modest favourable impact on findings; a 20% reduction in risk would reduce the cost per QALY to £10,195. In the unlikely event of a 100% risk reduction, the cost per QALY would fall to £7,973.

The model does not appear sensitive to other parameters, including the costs of providing treatment or likelihood of inpatient admission following self-harm. If the productivity losses of all inpatient stays were included at a maximum value of £100 per day, then the cost per QALY could fall to £9,460—compared with £9,980 when we valued each day at £65.40 using the national living (minimum) wage rate.

### Probabilistic sensitivity analysis

**Figure 3.**
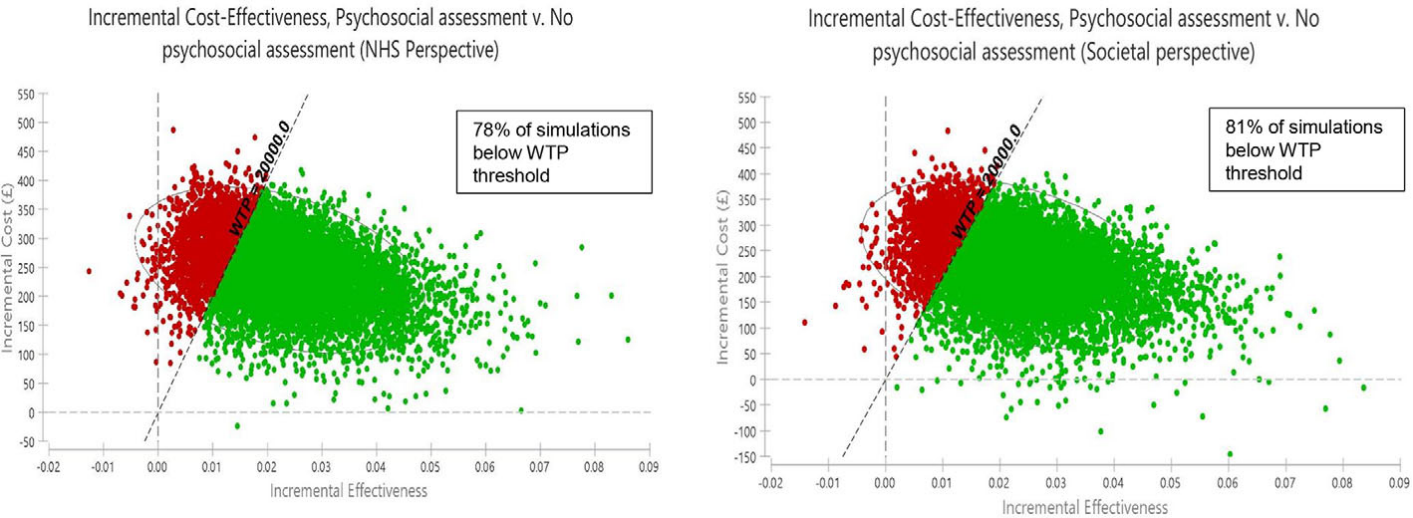
Cost-effectiveness planes (NHS and societal perspectives). Green dots represent simulations below the £20,000 per QALY gained cost-effectiveness threshold while red dots represent simulations that are above this threshold and are not considered cost-effective.

Cost-effectiveness planes showing results of probabilistic sensitivity analyses, with 10,000 simulated ICERs are shown in Figure 3. The elliptical circles included 95% of simulations in the model. From the NHS perspective, 78% of simulated pairs of costs and QALYs will have an incremental cost per QALY gained below £20,000. From our societal perspective, this increases to an 81% probability of psychosocial assessment having a cost per QALY gained below £20,000.

**Figure 4.**
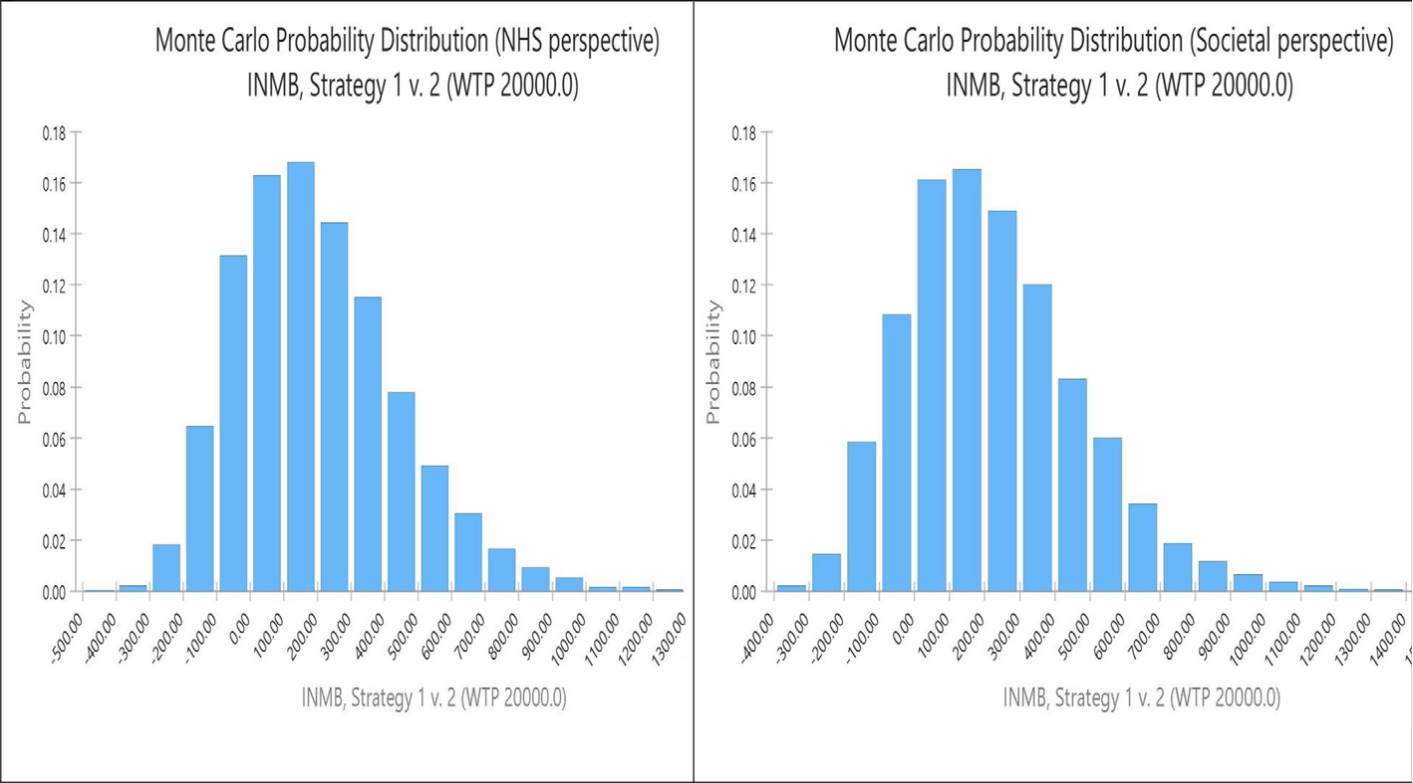
Net monetary benefit probability distribution (NHS and societal perspectives 10,000 bootstraps). Strategy 1, psychosocial assessment; Strategy 2, no psychosocial assessment.

**Figure 5.**
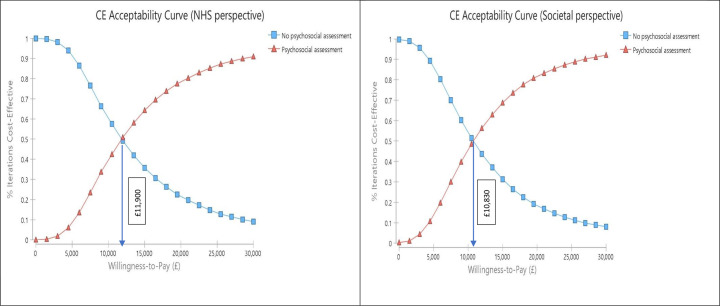
Cost-effectiveness acceptability curve (NHS and societal perspectives).

Figure 4 shows the distribution of NMB in probabilistic sensitivity analyses from the NHS and societal perspectives. In both cases, these are right-skewed with mean positive NMBs of £200 (95% CI: £189, £758) and £224 (CI: £180, £801) from the two perspectives, respectively. The cost-effectiveness acceptability curves in Figure 5 illustrate that use of psychosocial assessment has a higher probability of being cost-effective than no action if willingness to pay reaches £11,900 or £10,830 from the NHS and societal perspectives respectively. If a higher willingness to pay level of £30,000 per QALY gained was used, something considered appropriate where uncertainty is low [26], then the probability of intervention being considered cost-effective from an NHS perspective would rise to 91%. From a societal perspective at this higher threshold, the chance of being cost-effective would rise to 92%.

## Discussion

Although studies have not been designed to evaluate the effectiveness of psychosocial assessment, some suggest that assessment may be associated with reduced future risk of hospital-presenting self-harm. This may be partly due to receipt of more appropriate subsequent primary and community care, as well as therapeutic benefits of the psychosocial assessment process. This potentially is cost-effective and national clinical guidance on management of self-harm in England recommends psychosocial assessment should be offered to all individuals each time they present at hospital after self-harm [2,11]. Yet, there remains evidence of great variation in receipt of assessment in English hospitals [12,13,35].

We believe this is the first economic evaluation specifically on the benefits of increasing use of psychosocial assessment for all hospitals presenting self-harm. Our analysis suggests there is an economic case for adherence to NICE guidance, with a 78% chance from a health service perspective of cost per QALY gained being under the notional £20,000 threshold considered cost-effective by decision makers in England. Likelihood of psychosocial assessment being cost-effective was even higher when taking a partial societal perspective, even without incorporating additional impacts of reduced self-harm repetition on family members or productivity losses associated with completed suicide.

Our findings are consistent with the analysis of immediate impact of extending hours of a liaison psychiatry service at another English hospital, with a view to increasing the number of individuals who received psychosocial assessment [36]. After liaison psychiatry service hours were extended the proportion of individuals attending A&E following self-harm who received psychosocial assessment increased from 57 to 68%. The additional costs of liaison psychiatry were partly offset by a 14% reduction in hospital costs for self-harm management. That study also observed a plausible 20% reduction in future risk of hospital presenting self-harm, but was not sufficiently powered to detect any significant effect on self-harm rates. Analysis in Scotland of a very brief hospital administered psychological therapeutic intervention immediately after self-harm was also shown likely to be cost-effective, particularly for individuals with a history of self-harm [37].

Strengths of our modelling analysis include reliance on a large dataset across multiple hospitals where robust methods are used to identify all self-harm presentations regardless of whether these result in hospital admission [3]. We have also made use of detailed costing analyses from this dataset to estimate costs of psychosocial assessment and immediate treatment [27]. Conversely, because our model draws on data on self-harm presentations from a limited number of locations in England, these may not be representative of patterns of self-harm nationally, but our model was sensitive neither to changes in patterns of self-harm nor in rates of inpatient admission.

We also recognise that there appears, understandably, to have been no specific randomised controlled study looking solely at the role of psychosocial assessment in directly reducing future risk of self-harm. The magnitude of any therapeutic impact of psychosocial assessment on relative risk of repeat self-harm may vary considerably, depending, for example, on its quality. Our baseline probability of repeat self-harm after psychosocial assessment of 0.11 draws on data from two of the three hospital groups covered by the MSH that had a relative risk reduction of 0.59 following psychosocial assessment [14]. However, this might represent a “best case” scenario; our parameters might not be applicable to hospitals that, contrary to national guidance, only offer assessment to a minority of people who present with self-harm. In the third MSH hospital group, fewer than half of episodes resulted in assessment, with no discernible association with self-harm rates. Other studies have reported more conservative impacts. One study reported a relative risk of 0.7 for repeat self-harm, but this was based on experience in just one of the MSH, making its generalisability more limited [22]. Another English study using cohort data from three hospitals reported a relative risk of 0.82, but cautioned the estimate could be biased because of limitations in use of the instrumental variables methodology [21]. A previous modelling analysis, looking at “risk scales” as part of a hospital assessment process, assumed a relative risk of 0.8 for individuals receiving a psychosocial assessment [23]. In our sensitivity analysis, the results were most sensitive to changes in the probability of repeat self-harm following psychosocial assessment (Figure 2). This needs to be 0.13 or lower (equivalent to a relative risk of 0.73 for repeat self-harm) for the intervention to be considered cost-effective from a health system perspective at a cost per QALY threshold of £20,000.

It is also important to acknowledge, based on the earlier MSH study [14], that psychosocial assessment might be less effective, and therefore less cost-effective in the context of material deprivation and health inequalities. Further work to better understand these impacts and consider how a variety of clinical and social interventions in these settings to manage self-harm and to encourage engagement with services is needed. That said, if there was an increase of up to 20% in relative risk of self-harm for those with the highest levels of deprivation, in line with observations in the earlier study, our model indicates the intervention would still be cost-effective at £18,344 per QALY gained.

Considering how cost-effectiveness is linked to individual patient history merits future investigation. In our model, we have held the likelihood of each psychosocial assessment reducing the risk of future self-harm constant; evidence remains limited but differences in past patient history, including receipt of multiple psychosocial assessments, may improve the level of risk reduction [22]. Economic analyses could also look at the relative cost-effectiveness of measures to improve quality and/or fidelity to psychosocial assessment processes, especially for initial hospital presentations. To do this, however, more information is needed on the quality of the psychosocial assessment process, as well as content, skills, and formulation; this has not been a feature of previous analyses [38].

Our sensitivity analysis indicates the model is sensitive to assumptions about quality of life. Few studies have elicited quality of life values for hospital-presenting self-harm; we were able to use quality of life estimates collected as part of an English trial of family therapy following self-harm, but this study only examined quality of life of adolescents [28]. However, we identified another English study where quality of life scores following self-harm were lower, but that study only included adult psychiatric inpatients [39]. Conservatively, we have also not included impacts on quality of life or productivity losses of the family and friends of individuals who self-harm.

Our modelling analysis considered the impact on immediate assessment and treatment costs in hospital; future analyses need to examine impacts on longer-term contact with primary and specialist community mental health teams. Success in reducing self-harm and suicide will partly depend on the longer-term care and support individuals receive. Psychosocial assessment may increase the likelihood that individuals make use of appropriate services, and these costs have not been included. However, some of these costs are likely to be for the treatment of physical and mental health problems unrelated to self-harm behaviour. The extent to which these knock-on costs explicitly link with self-harm needs to be investigated.

This need for future work to look at long-term health service utilisation has also been highlighted in other comparable studies [36]. One previous English study looking at long-term health and social care service utilisation of a small number of individuals following an initial self-harm event suggests that continuity of care may be relatively modest; average cumulative costs per individual of over 7 years were £3,991 (2020 prices) [40]. This study also reported much higher costs for individuals with multiple repeat events. This suggests that if future risk of self-harm is reduced through psychosocial assessment, then we may have omitted future avoided costs through better self-harm management and underestimated the economic case for psychosocial assessment. Including long-term costs beyond the health sector would also strengthen the economic case, with one recent Canadian analysis pointing to substantial long-term impacts on lost lifetime earnings linked to self-harm [41].

## Conclusions

Psychosocial assessment as implemented in the English NHS is likely to be cost-effective. However, further evidence about the precise impact of psychosocial assessment on self-harm repetition, as well as quality of life benefits of self-harm avoidance and costs to individuals affected by self-harm and their families beyond immediate hospital stays, is still needed. Given variable adherence to guidelines in England, it is also imperative to explore the cost-effectiveness of mechanisms that could change health care professional practice to better improve adherence.

## Data Availability

Individual patient-level from the Multicentre Study of Self-Harm will not be available due to confidentiality and data-sharing agreements in place.

## Supplementary Materials

To view supplementary material for this article, please visit http://doi.org/10.1192/j.eurpsy.2022.5. click here to view supplementary material
