# Supplementary material for: Cost-effectiveness of psychosocial assessment for individuals who present to hospital following self-harm in England: A model-based retrospective analysis
Source: Eur Psychiatry. 2022 Jan 31;65(1):e16. doi: 10.1192/j.eurpsy.2022.5 (PMC8926908; doi:10.1192/j.eurpsy.2022.5)
Supplement: Supplementary file 1 [file S0924933822000050sup.zip › S0924933822000050sup001.docx]

**Supplementary Figure A1: Detailed excerpt of model structure**


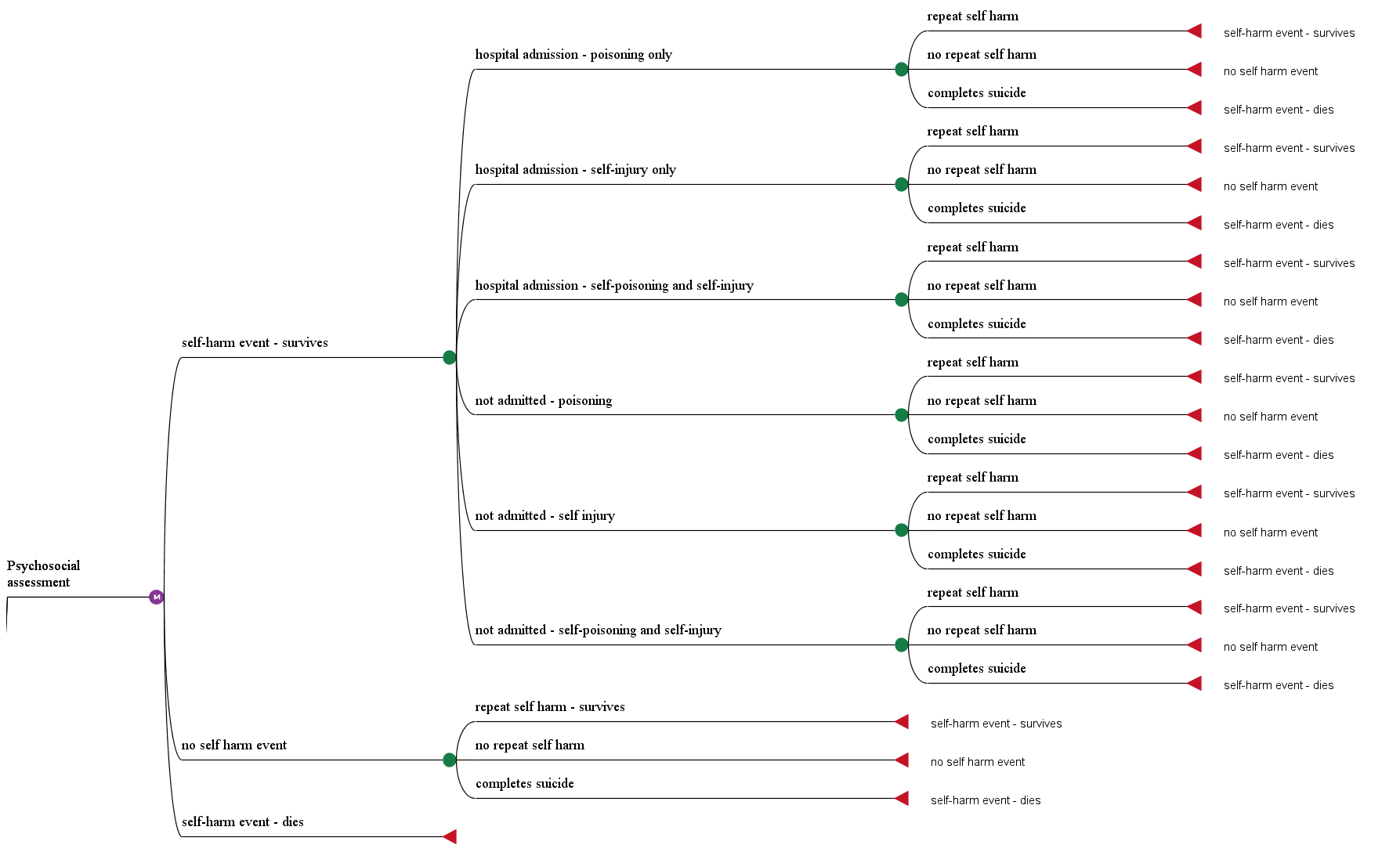


Note: Model structure is the same for the no intervention pathway (not shown)
